# Viral Proteins Originated *De Novo* by Overprinting Can Be Identified by Codon Usage: Application to the “Gene Nursery” of *Deltaretroviruses*

**DOI:** 10.1371/journal.pcbi.1003162

**Published:** 2013-08-15

**Authors:** Angelo Pavesi, Gkikas Magiorkinis, David G. Karlin

**Affiliations:** 1 Department of Life Sciences, University of Parma, I-43124, Parma, Italy; 2 Department of Zoology, University of Oxford, Oxford, OX1 3PS, United Kingdom; 3 The Division of Structural Biology, University of Oxford, Oxford, OX3 7BN, United Kingdom; University of Texas at Austin, United States of America

## Abstract

A well-known mechanism through which new protein-coding genes originate is by modification of pre-existing genes, e.g. by duplication or horizontal transfer. In contrast, many viruses generate protein-coding genes *de novo*, via the overprinting of a new reading frame onto an existing (“ancestral”) frame. This mechanism is thought to play an important role in viral pathogenicity, but has been poorly explored, perhaps because identifying the *de novo* frames is very challenging. Therefore, a new approach to detect them was needed. We assembled a reference set of overlapping genes for which we could reliably determine the ancestral frames, and found that their codon usage was significantly closer to that of the rest of the viral genome than the codon usage of *de novo* frames. Based on this observation, we designed a method that allowed the identification of *de novo* frames based on their codon usage with a very good specificity, but intermediate sensitivity. Using our method, we predicted that the Rex gene of *deltaretroviruses* has originated *de novo* by overprinting the Tax gene. Intriguingly, several genes in the same genomic region have also originated *de novo* and encode proteins that regulate the functions of Tax. Such “gene nurseries” may be common in viral genomes. Finally, our results confirm that the genomic GC content is not the only determinant of codon usage in viruses and suggest that a constraint linked to translation must influence codon usage.

## Author Summary

How does novelty originate in nature? It is commonly thought that new genes are generated mainly by modifications of existing genes (the “tinkering” model). In contrast, we have shown recently that in viruses, numerous genes are generated entirely *de novo* (“from scratch”). The role of these genes remains underexplored, however, because they are difficult to identify. We have therefore developed a new method to detect genes originated *de novo* in viral genomes, based on the observation that each viral genome has a unique “signature”, which genes originated *de novo* do not share. We applied this method to analyze the genes of Human T-Lymphotropic Virus 1 (HTLV1), a relative of the HIV virus and also a major human pathogen that infects about twenty million people worldwide. The life cycle of HTLV1 is finely regulated – it can stay dormant for long periods and can provoke blood cancers (leukemias) after a very long incubation. We discovered that several of the genes of HTLV1 have originated *de novo*. These novel genes play a key role in regulating the life cycle of HTLV1, and presumably its pathogenicity. Our investigations suggest that such “gene nurseries” may be common in viruses.

## Introduction

Modification of existing genes, such as by duplication or fusion, is a common and well-understood mechanism by which protein-coding genes originate [1], [2]. In contrast, we have shown that viruses generate many proteins *de novo* (hereafter called “*de novo* proteins”) [3], [4]. Preliminary observations indicate that these proteins play an important role in the pathogenicity of viruses [3], [5], for instance by neutralizing the host interferon response [6] or antagonizing the host RNA interference [7]. Strikingly, p19, the only *de novo* protein characterised both structurally and functionally, has both a previously unknown structural fold and a previously unknown mechanism of action [7]. Thus, protein innovation seems to be a significant, but poorly understood part of the evolutionary arms race between hosts and their pathogens [5], 8,9.

**Figure 1:**
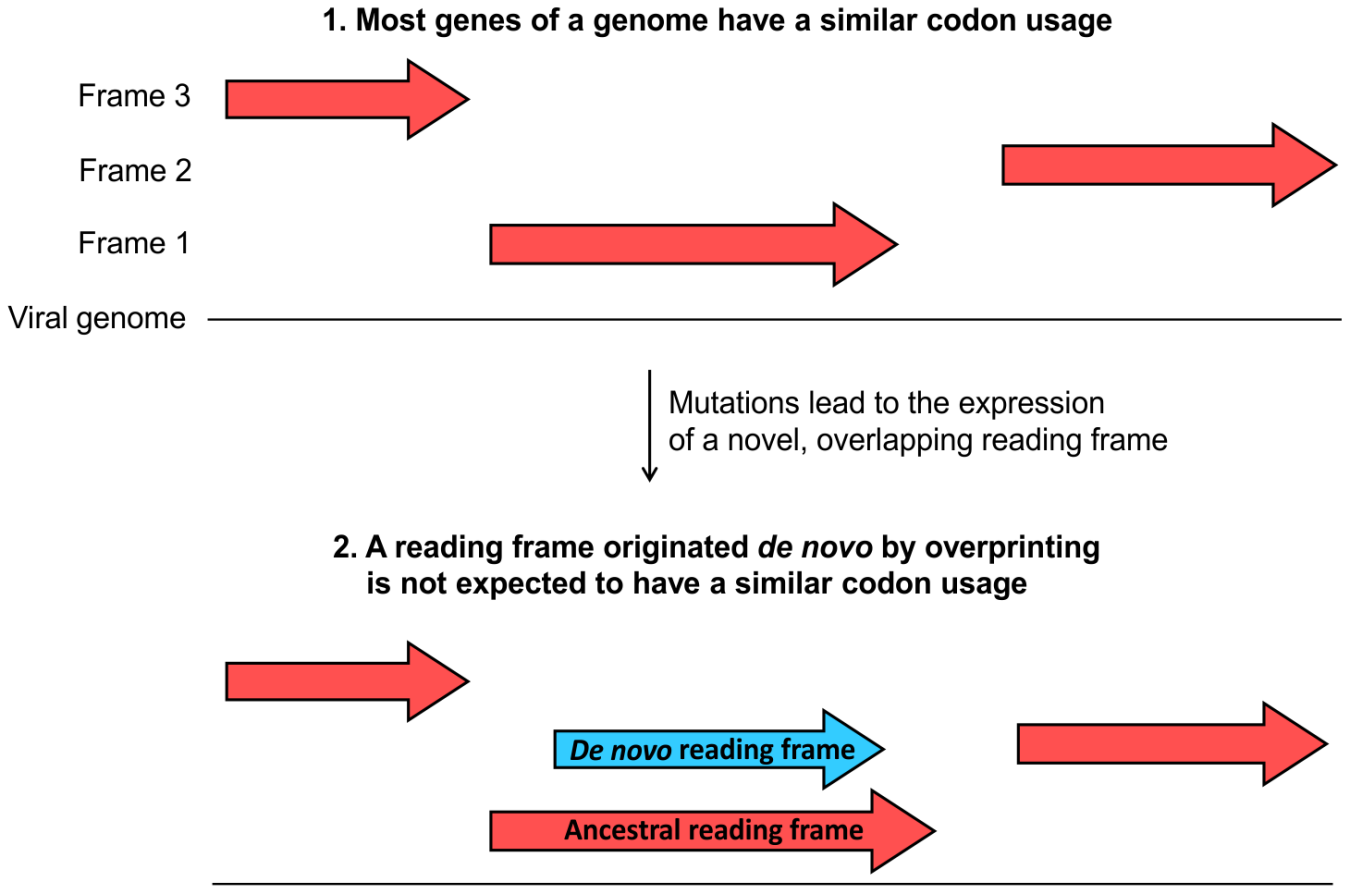
Rationale for our approach.

Studying *de novo* proteins should thus greatly enhance our understanding of host-pathogen co-evolution and our knowledge of the function and structure of viral proteins [3], [10]–[14]. However, a major bottleneck that prevents the study of such proteins is their identification, which is very challenging. Finding that a viral protein has no detectable sequence homolog does not reliably indicate that it has originated *de novo*, because viral proteins evolve so fast that they can easily diverge in sequence beyond recognition. To circumvent this problem, in our previous work [3], [4] and in the current study, we focused on a special case of *de novo* proteins: those generated by overprinting. Overprinting is a process in which mutations in a protein-coding reading frame allow the expression of a second reading frame while preserving the expression of the first one (Figure 1), leading to an overlapping gene arrangement [10]. It is thought that most overlapping genes evolve by this mechanism, and that consequently each gene overlap contains one ancestral frame and one originated *de novo* [10]. Because overlapping genes are particularly abundant in viruses [15]–[17], they constitute a rich source of *de novo* proteins.

Identifying which frame is ancestral and which one is *de novo* (the “genealogy” of the overlap) can be done, in principle, by examining their phylogenetic distribution (the frame with the most restricted distribution is assumed to be the *de novo* one). One can exclude the possibility that the phylogenetically restricted frame is in fact present in other genomes but has diverged beyond recognition, by checking that outside of its clade, the ancestral frame is not overlapped by any reading frame [4]. This approach is simple and reliable [3], [4], but is not applicable to cases where the homologs of both frames have an identical phylogenetic distribution. For instance, it could identify the *de novo* frame in only a minority (40%) of overlaps in our previous study [3]. Therefore, a new method is needed to identify the *de novo* proteins in most overlapping genes.

The approach we investigated is based on the hypothesis that the ancestral frame should have a pattern of codon usage (i.e. which synonymous codon(s) is preferred to encode each amino acid [18]) closer to that of the rest of the viral genome than the *de novo* frame [10]. Indeed, analyses of plant RNA viruses and animal DNA viruses [19], [20] have shown that, within a given viral genome, genes generally have a similar pattern of codon usage, which is thought to depend on the overall GC content of the genome [19]–[21]. In overlapping genes, the ancestral frame, which has co-evolved over a long period with the other viral genes, is expected to have a codon usage similar to that of the rest of the genome (Figure 1). On the other hand, the *de novo* frame, at birth, will have a codon usage in effect randomized by the shift and thus unlikely to be close to that of the genome. In addition, constraints imposed by the ancestral frame might prevent the *de novo* frame from adopting, later, the typical genomic codon usage. Consequently, the *de novo* frame is expected to have a codon usage less similar to that of the viral genome than the ancestral frame (Figure 1). This approach has been empirically used to try and identify the *de novo* frame in a number of cases, as have been related methods which rely on the frequency on nucleotides at some or all codon positions [10], [22]–[29]. However, the reliability or accuracy of these methods has never been tested. Here we gathered a reference (“benchmark”) dataset composed of overlaps with known genealogy, and used it to answer the following questions: do *de novo* frames have a codon usage distinguishable from ancestral frames? If yes, can codon usage be used to identify the *de novo* frame? What is the reliability of the method and its sensitivity? Finally, we applied this method to overlapping genes whose genealogy was undeterminable by the phylogenetic method.

## Results

### A benchmark dataset of overlapping genes with known genealogy

**Figure 2:**
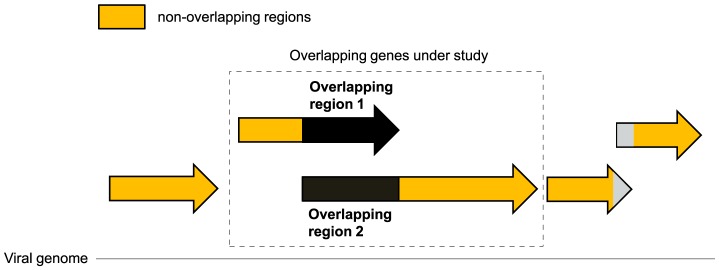
Definition used for overlapping regions and non-overlapping regions of a viral genome. If a viral genome contains other overlapping genes than those under study (e.g. the genes to the right), we only considered non-overlapping regions of these genes; their overlapping regions (in grey) were excluded from the analysis.

As described in Material and Methods, we assembled a dataset of 27 independent, experimentally proven overlapping genes longer than 140 nt (Table 1). 16 of them have been described previously [3], as indicated by an asterisk in Table 1, and 11 additional overlaps were collected for this study. The 27 overlaps come from 25 genera, distributed in 16 viral families covering a wide range of viruses (Table 1). 18 overlaps involve one gene being completely overlapped by the other, while in 9 the overlap is partial (e.g. Figure 2). All overlapping genes are in the same orientation, i.e. there are no antiparallel overlapping genes in the dataset. To identify the genealogy of the overlaps, we used the same stringent criterion as in our previous study [3], selecting only cases in which one frame, predicted ancestral, had a much wider taxonomic distribution than the other frame, predicted *de novo*. To be confident about the taxonomic distribution of each frame, we carried out extensive searches involving the most up to date similarity search tools, supplemented by in-depth manual searches using contextual information (see Material and Methods). The taxonomic distribution of each frame, and the corresponding evidence, are presented in Supplementary Table S1. Our predictions of ancestry are supported by functional data: almost all proteins encoded by a frame identified as ancestral have a function central to the viral cycle (such as capsid or replication), while most proteins identified as *de novo* have a “secondary” function related to pathogenicity (such as silencing suppressor or apoptotic factor) (Table 1). Thus, the predicted genealogy of the overlapping genes of the dataset is highly reliable.

**Table 1:** Benchmark dataset of 27 overlapping genes with known genealogy.

Viral family and nature of the genome	Genus and genome accession number(1) ^,^ (2)	Species	Ancestral frame [function(3)]	*De novo* frame [function(3)]	Length of overlapping regions (nt)	Length of non- overlapping regions (nt)	Host organism	Reference
*Alphaflexiviridae* ssRNA(+)	*Betatetravirus* (1) NC_001990	*Nudaurelia capensis beta virus*	Capsid protein [capsid]	Replicase, C-term domain	1833	3951	Insect	[106]
*Alphaflexiviridae* ssRNA(+)	*Mandarivirus* (1) NC_003093	*Indian citrus ringspot virus*	Capsid protein [capsid]	NABP, N-term domain	303	7011	Plant	[107]
*Alphaflexiviridae* ssRNA(+)	*Potexvirus* (1) NC_001658	*Cassava common mosaic virus*	TGBp2 [viral Movement]	TGBp3, N-term domain	153	5991	Plant	[108]
*Betaflexiviridae* ssRNA(+)	*Capillovirus* (1) NC_001749	*Apple stem grooving virus*	MP [viral Movement]	Polyprotein	966	5352	Plant	[109]
*Betaflexiviridae* ssRNA(+)	*Trichovirus* (1) NC_001409	*Apple stem leaf spot virus*	Coat protein [capsid]	MP, C-term domain	318	6804	Plant	[110]
*Birnaviridae* dsRNA	*Aquabirnavirus* (1) NC_001915	*Infectious pancreatic necrosis virus*	VP2 [capsid]	VP5 [apoptotic factor]	396	5112	Fish	[111]
*Bunyaviridae* ssRNA(−)	*Orthobunyavirus* (1) NC_001927	*Bunyamwera virus*	N [Nucleoprotein]	NSs [blocks interferon and cellular transcription]	309	11412	Insect	[112]
*Caliciviridae* ssRNA(+)	*Norovirus* (2) NC_008311	*Murine norovirus*	Capsid protein [capsid]	VF1 [virulence factor]	642	6639	Mammal	[113]
*Circoviridae* ssDNA	*Gyrovirus* (2) NC_001427	*Chicken anemia virus*	VP2 [phosphatase?]	Apoptin [apoptotic factor]	366	1275	Bird	[114]
*Dicistroviridae* ssRNA(+)	*Aparavirus* (2) NC_009025	*Israel acute paralysis virus*	Capsid Protein [capsid]	Pog	315	8115	Insects	[115]
*Geminiviridae* ssDNA	*Begomovirus* (2) NC_001467	*African cassava mosaic virus*	AL1 [rolling circle replication initiator]	AC4 [silencing suppressor]	426	2928	Plant	[116]
*Hepadnaviridae* Retroid	*Orthohepadnavirus* (1) NC_003977	*Human hepatitis B virus*	Pol, central domain [reverse transcriptase]	L [envelope glycoprotein]	708	1578	Mammal	[117]
*Hepadnaviridae* Retroid	*Orthohepadnavirus* (1) NC_003977	*Human hepatitis B virus*	Pol, C-term domain [RNAse H]	X [virulence factor]	252	1578	Mammal	[118]
*Luteoviridae* ssRNA(+)	*Luteovirus* (2) NC_003680	*Barley yellow dwarf virus*	P5 [capsid]	P4 [viral Movement]	468	4311	Plant	[119]
*Parvoviridae* ssDNA	*Brevidensovirus* (2) NC_004285	*Aedes albopictus densovirus*	NS1 [rolling circle replication initiator]	NS2(4) [virulence factor]	1122	2142	Insect	[120]
*Parvoviridae* ssDNA	*Densovirus* (2) NC_005041	*Blattella germanica densovirus*	NS1 [rolling circle replication initiator]	NS2(4) [virulence factor]	792	4005	Insect	[121]
*Parvoviridae* ssDNA	*Dependovirus* (2) NC_001401	*Adeno-associated virus 2*	VP2 [capsid]	AAP [capsid assembly co-factor]	618	3429	Mammal	[122]
*Parvoviridae* ssDNA	*Parvovirus* (2) NC_001718	*Porcine parvovirus*	Capsid protein [capsid]	SAT [virulence factor]	210	3639	Mammal	[123]
*Carmotetraviridae* ssRNA(+)	*Alphacarmotetravirus* (2) NC_014126	*Providence virus*	p104(5) [polymerase]	p130	2682	3312	Insect	[31]
*Tetraviridae* ssRNA(+)	*Omegatetravirus* (1) NC_005899	*Dendrolimus punctatus tetravirus*	Capsid protein [capsid]	p17	384	6591	Insect	[124]
*Tombusviridae* ssRNA(+)	*Carmovirus* (1) NC_003608	*Hibiscus chlorotic ringspot virus*	p28 [replicase cofactor]	p23 [virulence factor]	633	2163	Plant	[125]
*Tombusviridae* ssRNA(+)	*Carmovirus* (1) NC_003608	*Hibiscus chlorotic ringspot virus*	Capsid protein [capsid]	p25 [viral movement]	678	2163	Plant	[125]
*Tombusviridae* ssRNA(+)	*Tombusvirus* (1) NC_003532	*Cymbidium ringspot virus*	p22 [viral movement]	p19 [silencing suppressor]	522	3648	Plant	[126]
*Tombusviridae* ssRNA(+)	*Machlomovirus* (2) NC_003627	*Maize chlorotic mottle virus*	Capsid protein(5) [capsid]	p31	453	2625	Plant	[127]
*Tymoviridae* ssRNA(+)	*Tymovirus* (1) NC_004063	*Turnip yellow mosaic virus*	Replicase [Methyltransferase-Guanylyltransferase]	MP [viral movement]	1881	4230	Plant	[128]
*Virgaviridae* ssRNA(+)	*Hordeivirus* (1) NC_003481	*Barley stripe mosaic virus*	TGBp2 [viral movement]	TGBp3, N-term domain	192	8796	Plant	[129]
Unassigned ssRNA(+)	*Umbravirus* (1) NC_004366	*Tobacco bushy top virus*	ORF4 [viral movement]	ORF3 [viral movement]	699	2619	Plant	[130]

(1) gene overlaps described previously (see reference [3]).

(2) additional overlaps collected for this study.

(3) The function is that of the overlapping region of the protein; if it is not known, the field is left blank.

(4) The NS2 proteins of *brevidensoviruses* and that of *densoviruses* are not homologous (they are encoded in different frames relative to NS1).

(5) The *alphacarmotetravirus* polymerase and *machlomovirus* capsid have originated by horizontal transfer and thus the two corresponding overlaps are not part of the benchmark dataset, although we perform the same analyses on them than on other overlaps(see text).

Abbreviations: AAP, assembly-activating protein; dsRNA, double-stranded RNA; C-term, C-terminal; L, large envelope protein; MP, movement protein; NABP, nucleic-acid binding protein; NS, non-structural protein; NSs, non-structural protein of the small RNA segment; N-term, N-terminal; Pog, predicted overlapping gene; Pol, Polymerase; SAT, small alternatively translated protein; ssDNA, single-stranded DNA; ssRNA, single-stranded RNA (+, positive or −, negative); TGBp2, Triple Gene Block protein 2; TGBp3, Triple Gene Block protein 3; VP, viral protein.

We needed to exclude from the dataset ancestral frames that have entered their genome by distant horizontal transfer since these frames are not expected to have the same codon usage as that of their new viral genome, and are thus not suitable for codon usage analysis. Performing a detailed recombination analysis on all ancestral frames of the dataset was out of the scope of this article, and thus we simply detected cases of taxonomic incongruence (see Material and Methods). We detected two cases in which the ancestral frame had originated from another viral genome by distant horizontal transfer. The ancestral protein p104 of *Providence virus* (genus *alphacarmotetravirus*, family *Carmotetraviridae*) has statistically significant similarity with the replicase of viruses from a different family, *Tombusviridae*. Also, the capsid protein of *Maize chlorotic virus* (genus *machlomovirus*, family *Tombusviridae*) has significant similarity to that of *sobemoviruses*, an unassigned genus unrelated to *Tombusviridae* [30]. We established that horizontal transfer took place towards *alphacarmotetravirus* and *machlomovirus* from the other families by analysing the phylogenetic distribution of homologs of the ancestral proteins (not shown). Our results agree with previously reported findings that *Providence virus* has originated through recombination between a *Tombusviridae*-like and a *Tetraviridae*-like virus [31], and that the *machlomovirus* capsid protein is taxonomically incongruent [32]. We excluded these two cases from our analyses, and the final benchmark dataset is thus composed of 25 overlaps (Table 1).

### Ancestral frames have a codon usage closer to that of the genome than *de novo* frames

As a measure of codon usage similarity between a given frame and the rest of the viral genome, we used the Spearman's rank correlation coefficient (*r_s_*) between the number of occurrences of each codon in that frame and in the viral genome (see Materials and Methods). Accordingly, the higher the *r_s_* of a frame, the more similar its codon usage is to that of the genome. For all gene overlaps of the benchmark dataset, we evaluated *r_sA_*, *r_sN_* (the *r_s_* of the ancestral and the *de novo* frame, respectively), and the difference (*d*_21_) between *r_sA_* and *r_sN_* (*d*_21_ = *r_sA_*−*r_sN_*). They are listed in the left moiety of Table 2, ranked by decreasing value of t-Hotelling. *r_sA_* is higher than *r_sN_* in 21 cases (i.e. d_21_>0) and lower (i.e. d_21_<0) in only 4 cases. This distribution is not random (P<0.001, in accordance to the binomial proportion test), suggesting that ancestral frames have a codon usage closer to their genome than *de novo* frames. This conclusion is supported quantitatively, since the median *r_sA_* (0.42) is significantly (P<0.01) higher than the median *r_sN_* (0.19) according to the Wilcoxon signed rank test [33]. These findings support the hypothesis that codon usage can, in principle, be used to determine the ancestral frame.

**Table 2:** Analysis of the codon usage of overlapping frames from the benchmark dataset.

			Calculations performed on actual frames							Calculations performed on simulated frames				
Genus	Ancestral frame	*De novo* frame	N_A_	N_N_	r_sA_	r_sN_	*d_21_* = r_sA_−r_sN_	t-Hotelling	P<	r_sA_	r_sN_	*d_21_* = r_sA_−r_sN_	P<	Agreement between t-Hotelling and simulation
*Omegatetra*	Capsid	p17	49	48	0.70	0.04	0.66	4.26	0.001	0.252	0.239	0.012	0.001	Yes
*Noro*	Capsid	VF1	59	57	0.68	0.23	0.45	3.57	0.001	0.304	0.222	0.083	0.002	Yes
*Dependo*	VP2	AAP	59	57	0.61	0.19	0.42	3.03	0.005	0.312	0.255	0.056	0.001	Yes
*Carmo*	Replicase	p23	59	57	0.42	−0.10	0.52	3.00	0.005	0.305	0.245	0.060	0.001	Yes
*Aquabirna*	VP2	VP5	53	57	0.62	0.24	0.38	2.74	0.005	0.285	0.239	0.046	0.004	Yes
Luteo	P5	P4	59	52	0.44	0.01	0.43	2.72	0.005	0.314	0.284	0.030	0.0005	Yes
*Tymo*	Replicase	MP	57	59	0.79	0.59	0.20	2.65	0.01	0.294	0.292	0.002	0.073	No
*Capillo*	MP	Replicase	59	59	0.65	0.40	0.25	2.30	0.025	0.357	0.288	0.069	0.080	No
*Mandari*	Capsid	NABP	37	44	0.58	0.22	0.36	2.22	0.025	0.229	0.247	−0.019	0.011	Yes
*Carmo*	Capsid	p25	59	59	0.35	−0.05	0.40	2.13	0.025	0.316	0.227	0.089	0.003	Yes
*Betatetra*	Capsid	Replicase	59	59	0.40	0.11	0.29	1.88	0.05	0.306	0.290	0.017	0.003	Yes
*Gyro*	VP2	Apoptin	59	51	0.29	−0.01	0.30	1.78	0.05	0.259	0.217	0.042	0.020	Yes
*Potex*	TGBp2	TGBp3	20	33	0.42	0.03	0.39	1.75	0.05	0.237	0.206	0.031	0.015	Yes
*Parvo*	VP2	SAT	29	35	0.47	0.12	0.35	1.69	0.10	0.234	0.224	0.010	0.063	Yes
*Tombus*	p22	p19	59	59	0.33	0.13	0.20	1.24	0.15	0.310	0.289	0.021	0.051	Yes
*Apara*	Capsid	Pog	37	49	0.25	0.02	0.23	1.04	0.20	0.276	0.262	0.014	0.061	Yes
*Denso*	NS1	NS2	59	57	0.36	0.19	0.17	1.04	0.20	0.278	0.303	−0.024	0.083	Yes
*Orthohepadna*	Pol	L	59	55	0.42	0.29	0.13	0.98	0.20	0.241	0.252	−0.011	0.146	Yes
*Umbra*	ORF4	ORF3	59	55	0.40	0.23	0.17	0.97	0.20	0.280	0.279	0.001	0.101	Yes
*Begomo*	Replicase	AC4	53	55	0.18	0.10	0.08	0.49	0.50	0.317	0.281	0.036	0.377	Yes
*Hordei*	TGBp2	TGBp3	29	27	0.24	0.36	−0.12	0.39	0.50	0.195	0.209	−0.014	0.297	Yes
*Orthohepadna*	Pol	X	48	44	0.06	0.10	−0.04	0.24	0.50	0.221	0.217	0.004	0.438	Yes
*Brevidenso*	NS1	NS2	59	59	0.62	0.63	−0.01	0.09	0.50	0.348	0.353	−0.005	0.556	Yes
*Orthobunya*	N	NSs	55	55	0.28	0.26	0.02	0.06	0.50	0.308	0.235	0.073	0.655	Yes
*Tricho*	Capsid	MP	43	41	0.31	0.32	−0.01	0.03	0.50	0.313	0.278	0.035	0.426	Yes
Recombinant:														
*Alphacarmotetra*	Replicase	p130	59	59	0.00	0.51	−0.51	2.94	0.005	0.279	0.303	−0.024	0.001	Yes
*Machlomo*	Capsid	p31	43	41	0.34	0.18	0.16	1.01	0.20	0.273	0.230	0.044	0.189	Yes

Abbreviations are the same as in Table 1. The last two overlaps have entered their genome by horizontal transfer (see text).

*r_sA_* is the Spearman rank correlation coefficient *r_s_* between the codon usage of the ancestral frame and that of its genome. *r_sN_* is the equivalent coefficient for the *de novo* frame. N_A_ and N_N_ are the number of codons on which *r_sA_* and *r_sN_* were calculated. The first row indicates whether calculations are presented for the actual overlapping frames or for the corresponding simulated frames. The calculation of P for the actual frames is based on Hotelling's t-test, whereas for simulated frames P is based on the distribution of the simulated *d_21_* (see text). Agreement between t-Hotelling and simulation is calculated on the basis of whether corresponding P-values are both <0.05 or >0.05.

We now needed a method to infer, given any pair of overlapping frames, whether one frame had a codon usage significantly closer to the rest of the viral genome than does the other frame. In principle, a suitable method to assess the significance of the difference between the *r_s_* coefficients of each frame is Hotelling's t-test [34], [35]. However, Hotelling's t-test is applicable to correlation coefficients estimated from independent data, whereas our data are clearly not independent (see Material and Methods). Therefore, we assessed whether Hotelling's t-test was robust to the violation of the non-independence of data by comparing the results of Hotelling's t-test with simulated codon usage data (see Material and Methods). Values of *r_sA_*, *r_sN_* and d_21_ for simulated frames corresponding to each overlap are presented in the right moiety of Table 2. We performed a McNemar test [33], which indicated that both methods provide equivalent results (McNemar chi-square = 0.6; P = 0.50). Therefore, Hotelling's t-test is reasonably robust to violation of independence and is applicable to our problem.

### Codon usage can predict the ancestral frame with a high specificity but intermediate sensitivity

Having established the validity of Hotelling's t-test, we used it to predict the ancestral frame (and consequently the *de novo* frame) in our dataset. Given two overlapping frames 1 and 2, a frame (for instance frame 2) was predicted ancestral only if it matched the following two criteria:

1. its codon usage was significantly closer to that of the genome than the other frame, i.e. *r*_s2_>*r*_s1_ and the difference *d*_21_ = *r*_s2_−*r*_s1_ was statistically significant (P<0.05) according to Hotelling's t-test;
2. its codon usage was positively correlated to that of the genome, i.e. *r*_s2_>0.

The first criterion corresponds to our main biological hypothesis, whereas the second criterion avoids a scenario in which the first criterion gives results that are mathematically significant but not biologically meaningful. For instance, if one frame had an *r*_s_ of −0.7 and the overlapping frame had an *r*_s_ of −0.1, the difference would be significant. However, it would be unjustified to return a prediction that the second frame is ancestral, because the negative value of its *r*_s_ contrasts with our central hypothesis that the ancestral frame has conserved traces of the genome's codon usage.

The overlaps are listed in Table 3 by decreasing value of t-Hotelling. We found that both criterions were fulfilled for 13 of our 25 overlaps, and in all these cases the ancestral frame prediction was correct, i.e. concordant with that established by phylogeny (Table 3). Consequently, the specificity of the codon usage approach is high (specificity = 1.0, 95% confidence interval [CI] 0.77–1.00), but its sensitivity is moderate (sensitivity = 0.52, 95% CI 0.31–0.72).

**Table 3:** Prediction of the ancestral frame in overlapping genes from the benchmark dataset.

Genus	r_sA_	r_sN_	*d_21_* = r_sA_−r_sN_	t-Hotelling	P<	Predicted ancestral frame	Prediction correct?
*Omegatetra*	0.70	0.04	0.66	4.26	**0.001**	Capsid	Yes
*Noro*	0.68	0.23	0.45	3.57	**0.001**	Capsid	Yes
*Dependo*	0.61	0.19	0.42	3.03	**0.005**	VP2	Yes
*Carmo* (replicase/p23)	0.42	−0.10	0.52	3.00	**0.005**	Replicase	Yes
*Aquabirna*	0.62	0.24	0.38	2.74	**0.005**	VP2	Yes
*Luteo*	0.44	0.01	0.43	2.72	**0.005**	P5	Yes
*Tymo*	0.79	0.59	0.20	2.65	**0.01**	Replicase	Yes
*Capillo*	0.65	0.40	0.25	2.30	**0.025**	MP	Yes
*Mandari*	0.58	0.22	0.36	2.22	**0.025**	Capsid	Yes
*Carmo (capsid/p25)*	0.35	−0.05	0.40	2.13	**0.025**	Capsid	Yes
*Betatetra*	0.40	0.11	0.29	1.88	**0.05**	Capsid	Yes
*Gyro*	0.29	−0.01	0.30	1.78	**0.05**	VP2	Yes
*Potex*	0.42	0.03	0.39	1.75	**0.05**	TGBp2	Yes
*Parvo*	0.47	0.12	0.35	1.69	0.10	-	-
*Tombus*	0.33	0.13	0.20	1.24	0.15	-	-
*Apara*	0.25	0.02	0.23	1.04	0.20	-	-
*Denso*	0.36	0.19	0.17	1.04	0.20	-	-
*Orthohepadna* (pol/L)	0.42	0.29	0.13	0.98	0.20	-	-
*Umbra*	0.40	0.23	0.17	0.97	0.20	-	-
*Begomo*	0.18	0.10	0.08	0.49	0.50	-	-
*Hordei*	0.24	0.36	−0.12	0.39	0.50	-	-
*Orthohepadna* (pol/X)	0.06	0.10	−0.04	0.24	0.50	-	-
*Brevidenso*	0.62	0.63	−0.01	0.09	0.50	-	-
*Orthobunya*	0.28	0.26	0.02	0.06	0.50	-	-
*Tricho*	0.31	0.32	−0.01	0.03	0.50	-	-
Recombinant(1):						-	-
*Alphacarmotetra*	0.00	0.51	−0.51	2.94	0.005	p130	No(1)
*Machlomo*	0.34	0.18	0.16	1.01	0.20	-	-

(1) The last two overlaps have entered their genome by horizontal transfer and are not taken into account for calculations of specificity and sensitivity of the method.

Abbreviations and conventions are the same as in Table 2. A frame is predicted ancestral if its *r_s_* is positive and significantly higher than the *r_s_* of the other frame (P<0.05, corresponding to t-Hotelling >1.70). If no prediction is possible, the field is left blank. Numerical values are the same as in Table 3 for actual frames, but are reproduced here for clarity.

### Excluding possible confounding factors: genome segmentation, GC content, age, amino acid composition, relative frame, and recombination

We examined several factors that could influence the ability to predict the *de novo* frame by its codon usage.

A first factor is genome segmentation: five overlaps of the dataset belong to viruses with segmented genomes (*Aquabirnavirus, Begomovirus, Hordeivirus, Omegatetravirus, Orthobunyavirus*). The calculations above were done by considering all genomic segments of such viruses as their “genome”. However, considering only the segment encoding the overlap under study yielded the same predictions, suggesting that genome segmentation is not a confounding factor.

Second, an extreme GC content could also, in principle, confound codon usage analysis. However, the GC contents of the genomes we analysed here are in a moderate range (35–57%), and thus are probably not a source of bias.

Third, in principle, the relative frame (+1 or +2) of the *de novo* region with respect to the ancestral region could influence the power of codon usage analysis to distinguish their genealogy. As can be seen in Supplementary Table S2, 16 *de novo* coding regions are in the +1 frame relative to the ancestral region they overlap, while the remaining 9 *de novo* regions are in the +2 frame. Among the 13 overlaps for which there was a significant difference in codon usage between the two overlapping regions, in 9 cases the *de novo* region was in the +1 frame relative to the ancestral region, while in 4 cases it was in the +2 frame (Supplementary Table S2). A chi-square test (chi-square = 0.023; P = 0.90) indicates that the sensitivity of our method does not change depending on the relative frame of the *de novo* region with respect to the ancestral region, and thus that the relative frame is not a confounding factor.

A fourth factor is the age of overlaps: as *de novo* proteins age, they may progressively impose increased constraints on the ancestral frames, which may change their codon usage, and make it difficult or impossible to distinguish them from *de novo* frames [4]. Precisely estimating the age of overlaps is not possible given the state of our knowledge of viruses. However, one can use the taxonomical distribution of *de novo* frames as a heuristic to obtain a very approximate idea of their relative ages. For instance, a *de novo* frame found in a single species of viral family A has almost certainly appeared more recently than a *de novo* frame found in a whole family B (provided there is a good sequencing coverage in both families). We have applied this idea to infer the age of overlaps of the benchmark dataset.

*De novo* frames found only in one species were considered “young” (provided there are several species in the genus considered); overlaps found in more than one species but less than one genus were considered of “Intermediate” age, and overlaps found in more than one genus were considered “old”. We have indicated these estimated relative “ages” in Supplementary Table S2 (the exact taxonomic distribution of *de novo* frames is in Supplementary Table S1).

There is insufficient taxonomic coverage to estimate the age of overlaps in two genera, for which only a single species is known (*betatetravirus* and *mandarivirus*). The remaining 23 overlaps cluster in the following way: 3 young, 13 medium, and 7 old (supplementary Table S2). By codon usage analysis we have (correctly) predicted the genealogy of 3 young, 6 medium and 2 old overlaps (supplementary Table S2). We have analysed these data by the chi-square contingency table test. The Chi-square value was 1.95 (P = 0.30). Therefore, the predictive power of codon usage to identify the *de novo* frames does not seem to be dependent on their taxonomic distribution, and by extension, on their relative ages.

A fifth potential confounding factor is that some *de novo* frames have a biased amino acid (aa) composition. This raises the possibility that the aa composition of *de novo* frames could be the major explanatory factor of our results, and that differences in codon usage would be a consequence of this biased aa composition. To empirically determine whether aa composition contains more information about frame ancestry than codon usage, we carried out a correlation analysis of the aa composition of overlapping frames with the same statistical test as for codon usage, and compared the predictive power of both methods. We performed the same analysis as on codon usage data but on the frequency of the 18aas that have a degree of codon-degeneracy >1. The median value of the Spearman correlation between the aa composition of the ancestral frame and that of non-overlapping regions was 0.62, while the median value of the Spearman correlation between the aa. composition of the *de novo* frame and that of non-overlapping regions was 0.50. Unlike for codon usage (see above), the difference was not significant (P = 0.35 in accordance to the Wilcoxon signed rank test). Therefore, aa composition does not have as much predictive power regarding the genealogy of overlaps as codon usage, and our results are unlikely to be explained by the difference in aa composition between ancestral and *de novo* frames.

Finally, to study whether recombination could be a confounding factor, we examined codon usage in the two cases in which the ancestral frame had arisen by recombination (see above), excluded from the above statistics. For *machlomovirus*, the difference between *r*_s*N*_ and *r*_s*A*_ was not significant (Table 3, bottom, t-Hotelling = 1.01, P<0.20). On the other hand, in the case of *Providence virus (Alphacarmotetravirus)*, *r*_s*N*_ (0.51) was significantly higher than *r*_s*A*_ (0.00) (t-Hotelling = 2.94; P<0.005), and positive. Thus, ignoring the recombination event would lead to the erroneous prediction that the replicase is the *de novo* frame. It would be interesting to determine whether the codon usage of the *Providence virus* replicase gene is similar to that of its original genome. However, we could not find the species from which the recombination had occurred, since a similarity search based on the nucleotide sequence of the replicase found no similar viral (or cellular) sequence.

### Application of our method to cases irresolvable by the phylogenetic approach

We applied the codon usage method defined above to seven pairs of overlapping genes (gathered from the literature), in which both frames have the same phylogenetic distribution. Table 4 presents the codon usage values for these overlaps by decreasing value of t-Hotelling, and the corresponding predictions of ancestry. The codon usage of overlapping frames was significantly different in only two cases (*deltaretrovirus* Tax/Rex and *alphanodavirus* replicase/B2). *Deltaretrovirus* Tax and the *betanodavirus* replicase, respectively, had a codon usage significantly closer to that of the viral genome than the other frames, suggesting that they are the ancestral frames and that the *de novo* frames are Rex and B2. We discuss these two overlaps in more depth below (case studies number 1 and 2).

**Table 4:** Prediction, by codon usage, of the ancestral frame in overlapping reading frames with identical phylogenetic distribution.

Phylogenetic distribution	Genome accession number	Species	Frame 1 [function]	Frame 2 [function]	Length of overlap (nt)	Length of non-overlapping regions (nt)	r_s1_	r_s2_	t-Hotelling	P<	Predicted ancestral frame	Predicted *de novo* frame
Genus *Alphanodavirus*	NC_004142	*Boolarra virus*	Replicase A, C-term domain	B2 [silencing suppressor]	300	3930	0.62	0.16	2.74	**0.005**	Replicase A	B2
Genus *Deltaretrovirus*	HTU19949	*Human T-Cell lymphotropic virus 1*	Rex [post-transcriptional regulator]	Tax [transcription activator]	510	6021	0.32	0.58	2.06	**0.025**	Tax	Rex
Genus *Betanodavirus*	NC_003448	*Striped Jack nervous necrosis virus*	Replicase A, C-term domain	B2	231	3744	0.04	0.47	1.59	0.10	-	-
Genus *Ilarvirus*	NC_003842	*Tobacco streak virus*	Replicase, C-term domain	2b [silencing suppressor]	276	7338	0.32	0.26	0.37	0.50		
Genus *Polerovirus*	NC_001747	*Potato leafroll virus*	P0 [silencing suppressor]	P1, N-term domain	612	3789	0.30	0.24	0.38	0.50	-	-
Genus *Polerovirus*	NC_001747	*Potato leafroll virus*	P1	Replicase, N-term domain	456	3789	0.35	0.33	0.10	0.50	-	-
Genus *Cucumovirus*	NC_002035	*Cucumber mosaic virus*	Replicase, C-term domain	2b [silencing suppressor]	243	6900	0.14	0.11	0.12	0.50	-	-

Conventions are the same as in Table 3. A frame is predicted ancestral if its *r_s_* is positive and significantly higher than the *r_s_* of the other frame (P<0.05, corresponding to t-Hotelling>1.70).

In the five other overlaps analyzed in Table 4, both frames had a comparable codon usage, preventing prediction of the *de novo* frame.

### Case study 1: *De novo* origin of three *deltaretrovirus* accessory genes

**Figure 3:**
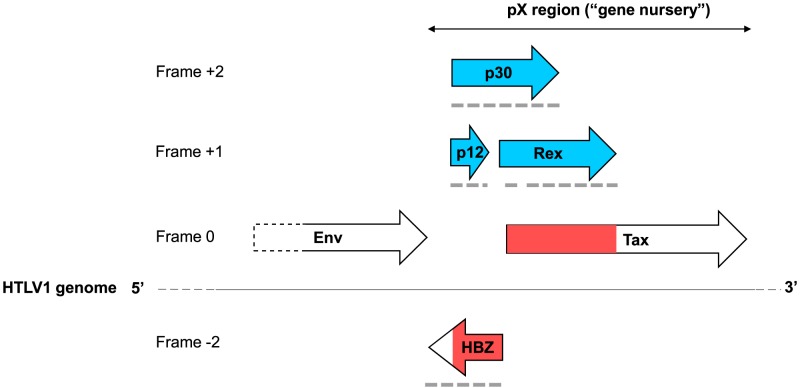
A “gene nursery”: the pX region of *deltaretroviruses*. The pX region of HTLV1 encodes five genes unique to *deltaretroviruses* by a complex pattern of alternative splicing and leaky scanning [36], [39]. The initial exons of these genes are very short and have not been represented, nor have been shorter versions of p12 and p30 expressed alternatively. Only the 3′ end of the Env gene is represented. The figure is approximately to scale. Ancestral regions in red and *de novo* regions in blue. Frame numbering is as in [45], with the Tax frame taken as “0”. Protein regions with unusually low sequence complexity are indicated by dashed, grey lines.

**Figure 4:**
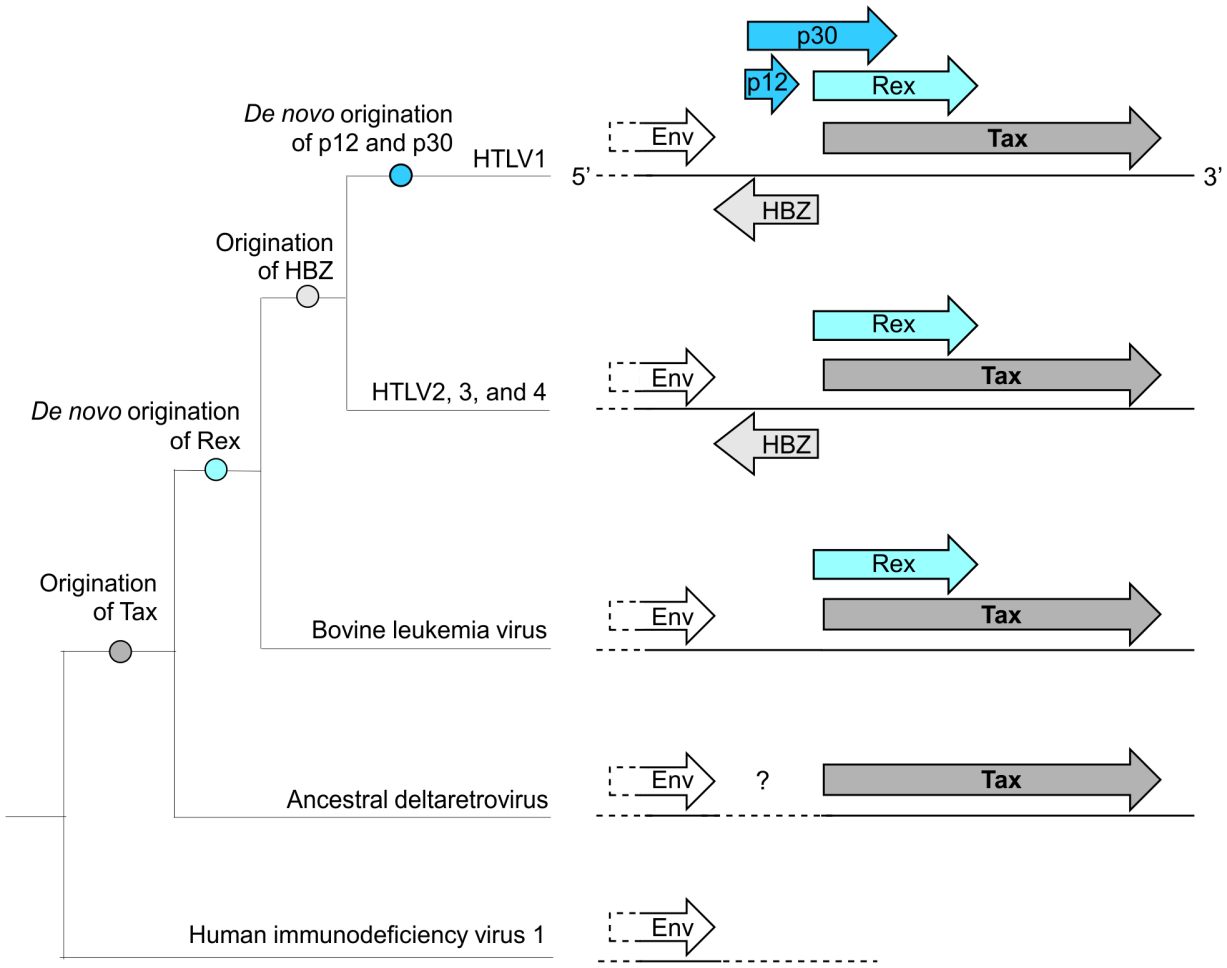
Presumed evolution of the *deltaretrovirus* pX region. The deltaretrovirus phylogeny is shown as a cladogram. Conventions are the same as in Figure 3.

We examined in more detail the *deltaretrovirus* genome, which contains a complex pattern of overlapping genes at its 3′ end, in the pX region [36]–[39]. In addition to Tax and Rex, the pX region of Human T-lymphotropic virus 1 (HTLV1) encodes two other proteins in the sense strand, p12 and p30, and a fifth protein, HBZ, from the antisense strand [36], [37], [40] (Figure 3). The resulting arrangement has two long (>80 aa) triple overlaps: the N-terminus of p30 overlaps both p12 and the N-terminus of HBZ, while the C-terminus of p30 overlaps the N-termini of both Tax and Rex (Figure 3). The phylogenetic distribution of the overlapping genes in *deltaretroviruses* is summarized in Figure 4. P30 is expressed only in HTLV1 [36]. p12 has only been reported in HTLV1 [36], and its coding sequence is interrupted by a stop codon in HTLV2, while it has no equivalent in *bovine leukemia virus*. HBZ is present in HTLV1 but also in HTLV2, 3 and 4, where it is called respectively APH2, APH3 and APH4 (these proteins have statistically significant similarity with HBZ, indicative of homology). In the bovine leukemia virus genome, no equivalent of HBZ is expressed from the antisense strand in the region between the Env and Tax genes (Luc Willems, personal communication); instead microRNAs are expressed from the sense strand [41], [42]. Taking into account this phylogenetic distribution, and our codon usage predictions, the most likely evolutionary scenario (Figure 4) is that HBZ has originated in the common ancestor of HTLV1 to 4, after its divergence from *bovine leukemia virus*; p12 has originated *de novo* in HTLV1 by overprinting HBZ; and p30 has originated *de novo* in HTLV1 by overprinting both HBZ (in the N-terminus of p30) and Tax and Rex (in the C-terminus of p30). It is not possible to conclude whether p30 or p12 originated first, nor how Tax or HBZ originated (*de novo* or by horizontal gene transfer).

We made two additional observations regarding codon usage. First, the fact that Tax and Rex are involved in a triple overlap with a short region of p30 (Figure 3) constitutes a potential confusing factor in our prediction of ancestry by codon usage above. Nevertheless, the region of p30 overlapping Tax and Rex has a codon usage significantly more distant to that of the genome than that of Tax (t-Hotelling = 2.16; P<0.025). Therefore, the codon usage of Tax is close to that of the genome over the entire length of its overlapping region, which further suggests that Tax is the ancestral gene. Second, genes expressed from an antisense strand are not expected to have a similar codon usage to genes from the sense strand. Accordingly, the codon usage of HBZ is not correlated to that of the rest of the genome (r*_s_* = 0.00 for the entire HBZ gene, r*_s_* = 0.06 for the region of HBZ overlapping p30).

The existence of triple overlaps poses severe constraints on the sequence of the proteins encoded by the pX region, and we thus examined whether they had an unusual sequence composition, or were predicted to be structurally disordered [3] (see Material and Methods). We found that all proteins encoded by the pX region, with the exception of Tax, contained long regions with low sequence complexity (as defined by SEG [43]) over most of their length (dashed lines in Figure 3; see Supplementary Table S3), indicating that they were unlikely to adopt a typical globular structure [43], [44]. Tax has no specific region of low sequence complexity, but both its N-terminus, overlapping Rex and p30, and its non-overlapping C-terminus have a highly biased composition, being enriched in hydrophobic residues (P<0.005) and depleted in polar and charged residues (P<0.005).

In addition, HBZ and Rex were predicted to be mostly disordered, at least in the absence of binding partners, while p30 contained several long regions predicted disordered (see Supplementary Table S3). Only p12 and Tax were predicted to be mostly ordered. These results suggest that sequence constraints imposed by triple overlaps may lead to proteins with a highly biased sequence composition and/or structurally disordered [3], and further highlight the fact that Tax is different from the other proteins encoded by the pX region.

Finally, it may seem extraordinary that triple overlaps exist at all, given the sequence constraints they impose; in that light, we note that the relative frame arrangement that would impose the highest constraint, called “−2” [45], is not used for the overlap involving HBZ. (In this arrangement, codon positions 1 and 2 of a frame overlap respectively codon positions 2 and 1 of the antisense frame, with the result that the sequences of each frame are almost fixed by each other). As can be seen in Figure 3, the frame that is in the −2 arrangement relative to HBZ is the non-coding frame 0, rather than the p12 or p30 frames.

### Case study 2: The B2 proteins of *Nodaviridae*, a complex evolutionary history

**Figure 5:**
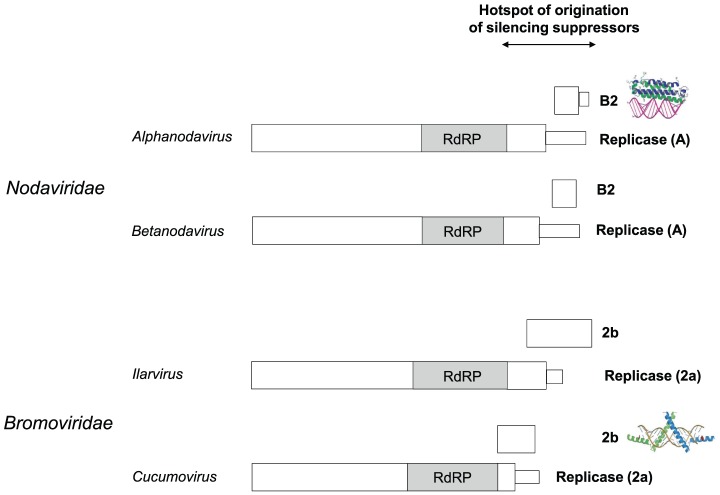
A genomic hotspot of origination of silencing suppressors in plus-strand RNA viruses. The replicases of *Nodaviridae* and *Bromoviridae* contain C-terminal extensions predicted disordered (thin boxes) downstream of their homologous polymerase (RdRP) domain. These extensions encode structurally unrelated suppressors of RNA silencing, B2 and 2b (PDB accession codes respectively 2AZ2 and 2ZI0) in different reading frames. Neither the C-terminal extensions nor the suppressors of RNA silencing have detectable sequence similarity, even between closely related genera. Which region is ancestral in each overlap could not be determined (see text).

In the second case, the codon usage of *alphanodavirus* B2 (a suppressor of RNA silencing [46]) suggests that it has originated *de novo* by overprinting the disordered C-terminal extension of the polymerase domain (Table 4). However, several observations cast a doubt on the reliability of this prediction. A similar genomic arrangement occurs in a closely related genus, *betanodavirus* (though there is no detectable sequence similarity between either the C-terminal extensions of the replicases or the B2 proteins of both genera) (Figure 5). A parsimonious scenario would demand that the overlaps of both genera have the same origin and thus presumably a similar codon usage pattern. Yet this is not the case: in *betanodavirus* it is B2 that has a codon usage closer to that of the genome (though not significantly so). This discrepancy might be due to horizontal transfer (see below).

Intriguingly, a very similar arrangement occurs in two genera (*ilarviruses* and *cucumoviruses*) of another family of positive-strand RNA viruses, *Bromoviridae*, in which a silencing suppressor called 2b overlaps a C-terminal extension of the polymerase (Figure 5). Like in *Nodaviridae*, neither the overlapping regions of the replicases nor the 2b proteins of the two genera have any similarity. The codon usage of the 2b proteins of *ilarviruses* and *cucumoviruses* is indistinguishable from that of the region of the replicase they overlap (Table 4), making a prediction of ancestry impossible. In fact, the 2b proteins of *ilarviruses* might have a different origin from those of *cucumoviruses*, since these genera do not form a monophyletic clade [47]. Despite their similar genomic location, size and function, *alphanodavirus* B2 and *cucumovirus* 2b have different structural folds and different modes of binding to RNA, both previously unknown [46], [48]–[50], clearly indicating an independent origin. We think that together, these observations indicate that the overlaps have a complex evolutionary origin; the ancestral protein could differ in each genus (for instance it might be the C-terminal extension of the replicase in *alphanodaviruses* and the B2 protein in *betanodaviruses*), and in some genera the ancestral proteins might have entered their genome by horizontal transfer (see below).

## Discussion

### Our method provides a quantitative improvement on previous empirical approaches

We have shown that *de novo* frames originated by overprinting have a pattern of codon usage distinguishable from ancestral frames, which can be used to predict the *de novo* frame with a good specificity but intermediate sensitivity (working in around half the cases).

How do our results compare with previous empirical studies of codon usage? The codon usage of six of the overlaps presented here has been studied previously using a different method, the “codon similarity index” [4]. The qualitative trends reported were similar to the ones we observe. Four of the overlaps presented here were also analysed previously, by Pavesi *et al* [24] who studied their information content and their codon usage. Again, the numerical values they reported for codon usage are in very good agreement with those obtained here, as are their general conclusions. However, our improved statistical analysis allowed us to draw more powerful conclusions. For instance, Pavesi *et al* reported that both the *tymovirus* replicase and movement genes had a codon usage correlated with that of their genome, and concluded that it was consequently not possible to determine the ancestral gene [24]. In the present article, the use of Hotelling's t-test to compare two dependent correlation coefficients [51] allowed us to determine that the replicase gene had a codon usage significantly closer to its genome than the movement gene (Table 3), indicating (correctly) that it was the ancestral frame. Another study, on the VP2/VP5 overlap of *avibirnavirus* (homologous to the *aquabirnavirus* overlap studied herein), showed that VP5 had an unusual nucleotide usage and predicted that it was the *de novo* frame [27]. Our quantitative analysis is in agreement with these findings: VP2 has a codon usage significantly closer to the viral genome than VP5 (Table 3). Finally, a previous analysis of the *cucumovirus* replicase/2b overlap predicted that 2b was the *de novo* frame, based on its uridine content at the third codon position [22]. In contrast, our analysis detects no statistically significant difference between the codon usage of the overlapping region of the replicase and that of 2b (Table 4).

### Limitations of our study

Why are ancestral and *de novo* frames distinguishable by their codon usage in only half of the overlaps? We examined in the Results several confounding factors, such as the relative frame of the overlapping regions, their sequence composition, and the estimated age of overlaps. They did not appear to have a significant impact on the predictive power of codon usage analysis. One note of caution is that we used a very crude estimate of the relative ages of overlaps (i.e. their taxonomic distribution). We could not use a more precise estimate, unlike a previous study [4], because our dataset contains both RNA and DNA viruses, which have no protein in common that could be used as a molecular clock, and because the proteins we studied often have more than 50% sequence divergence, preventing the determination of reliable phylogenies.

### Potential avenues for future research

During the revision of this manuscript, following the suggestion of a reviewer, we tested a distance measure based on information theory approaches: the modified Kullback-Leibler (KL) distance [52]. Since dinucleotide frequency is an important genome signature [53], we have re-analysed our dataset by calculating the KL distance (based on the frequency of the 16 dinucleotides at codon positions 1-2, 2-3, and 3-1) between the ancestral frame and the non-overlapping coding regions of the genome (KL_A_), and between the *de novo* frame and the non-overlapping coding regions of the genome (KL_N_). The ancestral frame had a KL distance to non-overlapping regions lower than that of the *de novo* frame in 23 out of 25 overlaps. In contrast, in our approach, the r_s_ of the *de novo* frame was lower than the r_s_ of the ancestral frame in 21 out of 25 overlaps.

We could not evaluate by analytical methods whether the KL distance between the ancestral frame and the non-overlapping regions (KL_A_) was significantly smaller than that of the KL distance between the novel frame and the non-overlapping regions (KL_N_), because KL distances are gamma-distributed [52] and there is no generic analytical solution for the distribution of the difference in gamma distributed variables. Therefore, instead, we performed, on each pair of overlapping genes from our dataset, a permutation test to estimate whether the observed (KL_N_−KL_A_) was significantly higher than the null distribution of (KL_N_−KL_A_) derived from 10,000 random permutations of the nucleotide sequence of the ancestral and the novel frame. We found that, on our dataset, this permutation test on the KL distance has the same specificity as the t-Hotelling test (1.00) and a slightly better sensitivity (0.60) than the t-Hotelling test (0.52), although the performance of the two methods is not significantly different (McNemar chi-square = 0.16; P = 0.70). We hope that our publicly available dataset of overlapping genes with known genealogy (expected to grow) will encourage others to continue exploring these methods and others.

### *Deltaretrovirus* Rex has probably originated *de novo* by overprinting Tax

Our new method allowed us to make predictions of ancestry for two overlaps in which both frames have the same phylogenetic distribution (Table 4). In the *alphanodavirus* replicase/B2 overlap (case study 2), several elements suggest that horizontal transfer might have taken place and thus that the codon usage prediction is not reliable. In the *deltaretrovirus* Tax/Rex overlap (case study 1), our prediction that Rex has originated *de novo* by overprinting Tax is consistent with the function of Tax, which occurs upstream of that of Rex in the viral cycle [37], [39], [54]. It is also coherent with the fact that Tax has a higher sequence complexity than Rex or p30, and is under stronger selection pressure than Rex [55], [56], which is generally the case of ancestral frames [3], [4]. Our prediction is in agreement with that of a previous work, reached by analyzing the substitution rates of Tax and Rex, their nucleotide composition and their amino acid composition [55]. Tax and Rex are encoded by the same mRNA but have different start codons [57] and thus Rex presumably originated by the appearance of a new ATG upstream of Tax. Both Tax and Rex are present in all *deltaretroviruses* and only in those viruses, which suggests that Tax originated first in the common ancestor of *deltaretroviruses*, and that Rex originated by overprinting it “rapidly” afterwards (by biological timescales), before the divergence of *deltaretroviruses*. Rex must have then undergone a rapid functionalization, since it is indispensable for the viral cycle, like Tax [37], [39], [54].

An alternative scenario is possible but appears much less parsimonious: Rex might have originated in another organism with a different codon usage, and entered the genome of the ancestor of *deltaretroviruses* by horizontal transfer. It would then have diverged in sequence beyond recognition, and have been overprinted by Tax (which would have a codon usage similar to that of the genome by coincidence).

### Hotspots of *de novo* origination or of horizontal transfer (“gene nurseries”) in viral genomes?

The pX region encodes five genes unique to *deltaretroviruses* [36]–[39], at least three of which (p12, p30 and Rex) have originated *de novo*, while the two others (Tax and HBZ) have either also originated *de novo* too (although earlier), or by horizontal transfer (Figure 3). The pX region thus constitutes a hotspot of gene origination, or gene “nursery” [58]. Strikingly, the two genes that have overprinted Tax, Rex and p30, play roles that are respectively complementary and antagonistic to Tax [38], [39], [59], while HBZ plays a role antagonistic to that of Tax [60], [61]. This suggests that the function of Tax was gradually controlled and refined by the appearance of new genes encoded in the same genomic location. Interestingly, other gene nurseries are found in a similar genomic position in other *Retroviridae*, such as *lentiviruses* or *spumaviruses* [62]. As seen above, the 3′ end of the replicase gene of some positive-strand viruses may also favour the origination of gene encoding silencing suppressors (Figure 5).

Such hotspots of origination of genes coding for proteins involved in the same pathways, and combining horizontal transfer and *de novo* origin, may be common in viruses. For instance, the movement proteins of *Alphaflexiviridae* and *Betaflexiviridae* are encoded in the same genomic position (downstream of the replicase gene) but belong either to the Triple Gene Block type [63], [64] or to the 30K type [65], indicating that at least one or possibly both types of movement proteins have entered these families by horizontal transfer [66].

The mechanisms that presumably favour the appearance of gene nurseries are unknown, but obviously of great interest. In the case of the *deltaretrovirus* pX region, we note that the high constraints imposed by the triple overlaps severely restrict the evolution of p12, p30 and Rex, and that consequently their present-day sequence composition is probably rather similar to the one they had when they first originated. We speculate that the pattern of origination seen in the pX region, in which *de novo* genes regulate the function of an ancestral protein, may be facilitated by the fact that low sequence complexity (and/or structural disorder) is compatible with a range of regulatory functions [67]–[69]. Thus, at birth, despite having a very “simple” sequence not honed by natural selection, these proteins may have had, by chance, a regulatory function and provided the virus with a fitness advantage that lead to their fixation.

### The need to annotate the genomes of *Retroviridae*, and to look for triple overlaps

*Retroviridae* encode numerous short, accessory genes, often accessed by alternative splicing or complex mechanisms leading to partially overlapping genes, and no doubt many remain to be discovered [62]. Yet at the time this article was submitted, almost none of these genes were annotated in the NCBI reference genomes [70]. This poor annotation is prejudicial to the study of these viruses. It is important that researchers who discover, or have discovered new genes, contact the NCBI viral genomes team to ensure that they are annotated properly.

Another, more general implication for genome annotation is that long, triple overlaps may have the potential to yield functional proteins relatively easily. Therefore, triple overlaps might be more abundant than previously thought (we know only two triple overlaps outside of *deltaretroviruses*, involving the P, V, and D or W proteins in *Paramyxovirinae* [71]–[74]). We thus recommend re-investigating known overlapping gene pairs to find whether in some cases a third overlapping frame might be expressed.

### A virus-specific evolutionary constraint that operates on top of GC mutational bias and influences codon usage

It has been proposed that the GC content of a genome was the main, though not the only, determinant of codon usage [19]–[21]. Our results confirm that it cannot be the unique determinant, otherwise the *de novo* and ancestral frames (which have the same GC content) would necessarily have a similar codon usage. Therefore, a significant evolutionary constraint(s) on codon usage must operate in addition to the GC content, and this constraint must be greater on ancestral frames than on *de novo* frames. Belalov *et al.* recently reported that the frequency of the dinucleotide CpG was an important factor of viral codon usage, and that CpG was significantly rarer at codon positions 2-3 than at positions 3-1 [75]. CpG is known to be underrepresented in RNA viruses [76], perhaps to avoid recognition from an antiviral CpG sensor [77]. However, the difference in frequency of CpG at different codon positions suggests that a second type of pressure exists, imposed by the translational apparatus. The authors thus suggested the existence of an evolutionary constraint on the genome deriving from a hypothetical cellular CpG sensor coupled (by an unknown mechanism) to the translational machinery. The existence of such a constraint would be coherent with our results, and we speculate that it might cause the difference in codon usage between ancestral and *de novo* frames.

### Conclusion

Very little is known about *de novo* protein origination, although it is by now clear that this mechanism plays an important role in viral pathogenicity. Our method should allow the identification of more *de novo* proteins, and thus enhance our understanding of host-pathogen co-evolution. It will be of particular interest to apply it to gene “nurseries” such as the ones we have identified here, and to elucidate the pressures that shape them. Finally, we note that recent experimental and computational reports suggest that *de novo* origination of genes by overprinting may not be confined to viruses but on the contrary, be a much wider phenomenon than previously thought, both in eukaryotic [78]–[82] and bacterial genomes [83].

## Materials and Methods

### Sequence analyses

We retrieved all sequences from the NCBI viral genome database [84]. We used MAFFT [85] for multiple sequence alignment, HHpred [86] and HHblits [87] for remote homology detection, Phylogeny.fr [88] for phylogenetic analyses, and METAPRDOS [89] for prediction of protein structural disorder, respecting the guidelines of [44]. We used Composition Profiler [90] for analyses of protein *global* compositional bias with respect to Swiss-Prot (release 51), and SEG for analyses of protein *local* compositional bias [43]. SEG analyses were obtained from the web server ANNIE [91] with parameters 45/3.75/3.4 in order to identify long regions with a composition bias indicative of non-globular proteins [44].

### Assembly of a benchmark dataset of overlapping genes with known genealogy

We searched the NCBI genome database [84] for viruses that infected eukaryotes, with a genome shorter than 30,000 nucleotides, and which contained overlapping genes longer than 120 nucleotides. The cut-off of 30,000 nucleotides was chosen because curation of larger genomes is impractical [3]. We included an overlapping gene into the benchmark dataset only when two criteria were fulfilled: 1) the expression of both overlapping reading frames was experimentally verified; 2) the genealogy of the overlapping reading frames could be determined with good support by using the very stringent criterion described previously [3], regarding the taxonomic distribution of both overlapping frames. According to this criterion, one reading frame can be considered ancestral only if it has homologs in at least two viral families whereas the other, overlapping frame had in at most one viral family. Since viral proteins diverge very fast, identifying viral proteins conserved in at least two families requires powerful similarity search techniques, which are described below. The final dataset, presented in Table 1, contains 27 independent (non-homologous) overlapping genes whose genealogy is reliably established. The dataset contains no antiparallel overlapping genes because we could not find any whose existence had been convincingly proven experimentally in the genomes of short or medium size considered (<30 kb).

### Definition of the boundaries of overlapping regions

We used the following conventions to define the precise boundaries of the overlapping regions on which we performed calculations of codon usage. There are two types of overlaps: in *internal overlaps*, one overlapping gene is contained entirely within the other gene whereas *terminal overlaps* involve only the 3′ end of one gene and the 5′ end of another [92]. In the case of internal overlaps, for the longest frame, the first codon counted as overlapping was the most upstream codon that overlaps the start codon of the internal frame, and the last codon counted as overlapping was the most downstream codon that overlaps the stop codon of the internal frame. In the case of terminal overlaps, for the upstream frame, the first codon counted as overlapping was the most upstream codon that overlaps the other frame, and for the downstream frame the last codon counted as overlapping was the most downstream codon that overlaps the stop codon of the other frame.

### Remote homology detection

In order to obtain a highly reliable genealogy of the overlaps, we needed to identify as distant homologs as possible for each protein of the dataset. However, not all homologs of a protein can be detected by conventional sequence similarity searches even if they have retained some sequence identity with the query, for a number of reasons [93], including the fact that databases of protein domains are underrepresented for viruses (our observations). We thus exploited “contextual” information available for viral proteins, such as taxonomy and genome organisation, to identify distant homologs overlooked by conventional searches [94]. We proceeded in the following way (the procedure is the same as in our previous article [3] but had not been described in detail). We first identified “straightforward” homologs of the query protein in the NCBI nr database (release 1st April 2012), by using HHpred [95] and HHblits [87] and selecting hits whose E-value was below the standard cut-off of 10^−3^. We then examined subsignificant hits (i.e. hits with an E-value superior to 10^−3^) up to E-values of 1000, looking for viral proteins that came from a taxonomically related virus, and/or occurred in the same position of the genome. Such subsignificant hits, which have weak similarity with the query protein and occur in a similar genomic context, constitute *potential* homologs. In order to test whether they were *actually* homologous with the query, we gathered homologs of these subsignificant hits (with E≤10^−3^), and used HHalign [96] to compare homologs of the query protein (obtained above) with homologs of the subsignificant hits. We considered that an HHalign E-value inferior to 10^−3^ indicated homology between the subsignificant hit and the query, but performed additional checks, such as verifying that the secondary structure and function (when available) of the hits were compatible with that of the query.

Whenever the structure of a protein from the dataset was available, we also performed structural similarity searches to identify structural homologs, using DALI [97] and FATCAT [98].

Because overlapping genes are not systematically recognised [16], [99] there is a theoretical possibility that some homologs of an overlapping frame might exist in related genomes but not be annotated, and therefore missed by similarity searches. For each overlap, we thus systematically checked that the genomes of other taxonomically related viruses did not contain conserved, unannotated open reading frames, as in [4].

We present in Supplementary Table S1 the taxonomic distribution of the homologs detected by our searches, together with the corresponding PFAM family (or clan) identified in the process.

### Detection of horizontal transfer events

Genes that have entered their genome by horizontal transfer can be identified by the fact that their phylogeny is discordant with the rest of the genome. A robust measure of this discordance is taxonomic incongruence, e.g. the existence of close homologs in a distant taxon. To detect taxonomic incongruence, we collected homologs of the protein products of each ancestral reading frame using blastp [100] on the Refseq database [101] with a cutoff E-value of 10^−3^. Hits to proteins from a different viral family than that of the query indicated taxonomic incongruence. To infer the direction of horizontal transfer, we analysed the phylogenetic distribution of homologs of the ancestral protein, both from the same family and from the distant taxon detected, and applied a parsimony criterion: the clade that has the wider phylogenetic distribution of the gene was most likely to be the clade of origin.

### Calculation of codon usage

In the genetic code, 18 amino acids (aas) are degenerate, e.g. encoded by more than one codon, and they are encoded by 59 “synonymous” codons in total. For each viral genome sequence, we measured the number of occurrences of the 59 synonyms in the non-overlapping coding regions and in each of the two overlapping reading frames (Figure 2). For clarity we will refer to the ensemble of the numbers of occurrences of the 59 synonymous codons of a given reading frame as its “codon usage”. The codon usage of non-overlapping regions will be called the “codon usage of the genome”.

In some overlapping reading frames (generally short, i.e. less than 400 nucleotides), the number of occurrences of the synonymous codons for a given aa was smaller than the degree of degeneracy of this aa (for instance only 3 synonyms for arginine, a 6-fold degenerate aa). In these cases, we restricted the analysis to synonymous codons whose number of occurrences was at least equal to the degree of degeneracy of the encoded aa. We indicated in Table 2 the number of synonymous codons on which the analysis was carried out.

### Preliminary tests on canonical methods of codon usage analysis

We wanted to utilize codon usage as a method to predict the genealogy of overlapping genes, and not simply to characterise the behaviour of overlapping genes. Therefore, we needed a method to assess whether the codon usage of ancestral frames was closer to the rest of the genome that the codon usage of *de novo* frames, and to assess whether this difference was statistically significant.

We have examined various canonical methods to evaluate codon usage bias: the Effective number of codons (ENC), [102] Codon Adaptation Index (CAI) [103], and Dmean index [104]. We found that ENC and Dmean had poor predictive power on the genealogy of overlaps (not shown). Initial tests suggested that CAI may have been more sensitive, but we could not easily test the statistical significance of the difference between the observed CAI distances. Therefore, we developed a new method, that had a good predictive power and that could yield estimates of statistical significance.

### A measure of the similarity in codon usage between two reading frames

Our hypothesis was that, in overlapping reading frames, the ancestral frame could be identified by having a codon usage that was more similar to the codon usage of the genome than that of the other frame. Thus we designed a measure of the similarity of codon usage of each frame with that of the genome, and a method to assess whether one frame had a codon usage significantly closer to that of the genome than the other frame.

In order to quantify the similarity between the codon usages of two given reading frames, we used as a measure the Spearman's rank correlation coefficient (*r*_s_) [33] between the number of occurrences of the 59 synonymous codons of these two frames (i.e. between their “codon usages”, see above). Each viral genome was divided into three sets: a) the overlapping region of the reading frame 1; b) the overlapping region of the reading frame 2, and c) non-overlapping regions of the genome, composed of the sequences of non-overlapping genes, and, in cases where some genes of the genome partially overlapped, of their non-overlapping regions (Figure 2). For viruses with segmented genomes, all segments were included in the calculations. For simplicity, the codon usage of the third set, i.e. non-overlapping regions, will be referred to as the “codon usage of the genome”. In all viral genomes, we calculated the *r*_s_ between the codon usage of the genome and that of each of the two overlapping frames under consideration (*r*_s1_ and *r*_s2_). The reason we collected the non-overlapping coding regions of a virus genome into an integrated set (as opposed to studying individual non-overlapping genes and analyzing their variance) is because the individual non-overlapping genes (or their non-overlapping regions, in cases of genes that partially overlap) are often short, which would have restricted correlation analysis to 2 or 3 dozens of synonyms.

### Assessing whether one of the two overlapping frames has a codon usage significantly closer to that of the genome

Determining if a given frame “1” has a codon usage closer to that of the genome than the other frame “2” is equivalent to determining whether *r_s1_* is significantly greater than *r_s2_*, i.e. whether the correlation between the codon usage of the first frame and that of the genome is significantly greater than the correlation between the codon usage of the second frame and that of the genome. This comparison involves two correlations coefficients that refer to a common variable (the codon usage of the genome), a situation categorized as “dependent correlation” [51] or as the study of “correlated correlation coefficients”, which can be addressed by the Hotelling t-test [34], [35]. The conventional Hotelling formula involves comparing Pearson correlation coefficients *r*_p_, but can be used with Spearman's correlation coefficients *r*_s_ by converting them into their Pearson equivalents: 
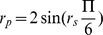
 [105].

The Hotelling t-value was calculated as follows:
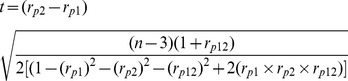
where *n* is the number of the compared codon frequencies, *r_p1_* and *r_p2_* are respectively the Pearson equivalents of *r_s1_* and *r*_s2_, and *r_p12_* is the Pearson equivalent of *r_s12_* (codon usage correlation between the overlapping frames). We assess the Hotelling t-value according to the one-tailed Student's t-test.

### Evaluation of the robustness of Hotelling's t-test to non-independence, by simulation

The Hotelling's t-test is designed for correlation coefficients estimated from independent data. However, the data we examine in this study (the number of occurrences of synonymous codons) are clearly not independent, since the sum of the numbers of synonymous codons encoding a given aa is fixed. Consider, for example, a reading frame containing 28 Glutamine codons (an aa encoded by two synonyms, CAA and CAG). If the number of occurrences of CAA is 11, that of CAG will inevitably be 17 (i.e. 28−11), i.e. the number of occurrences of CAA and CAG are not independent. Therefore, we assessed whether Hotelling's t-test was robust to non-independence of data by comparing it with a simulation-based exact test. For each pair of overlapping frames of the dataset, we generated two simulated overlapping frames with an aa composition identical to that of the two original frames, and used the actual non-overlapping regions of the genome as a reference set.

One round of simulation was performed as follows: we randomly generated a number (n) of occurrences for each of the 59 codons encoding the 18 degenerate aas, keeping the sum of the occurrences of codons encoding each aa equal to that of the original frame (e.g. if there were 28 Glutamine codons in the original frame, the simulated frame could have any number of CAA and CAG totalling 28). We calculated the correlation coefficients *r_s_*_1_ and *r_s_*_2_ between the number of occurrences of all synonyms in both simulated frames and that of the actual genome. We repeated the same process 10,000 times, thus simulating the distribution of *d*_21_ expected assuming that the reading frames are randomly generated and that codon usage is not related to ancestry (i.e. the null distribution). We then tested whether the observed d_21_ (Table 2) was significantly larger than this null distribution.

Finally, we used the McNemar's non-parametric test [33] to determine whether the Hotelling's t-test and the simulation provide equivalent results (which would indicate that the Hotelling's t-test is robust to non-independence of data).

## Supporting Information

Table S1 **Taxonomic distribution of the ancestral and de novo frames of the benchmark dataset.**
(DOC)

Table S2 **Examination of possible confounding factors of codon usage analysis: relative frame of** ***de novo*** **genes compared to ancestral genes, and relative age of de novo frames.** (1) We used taxonomic distribution as a very approximate, empirical proxy to estimate comparative ages of the overlaps. *De novo* frames found only in one species are considered “young” (provided there are several species in the genus considered, see note 2 below); overlaps found in more than one species but less than one genus are considered of “Intermediate” age, and overlaps found in more than one genus are considered “old”. The taxonomic distribution of *de novo* frames is taken from Supplementary Table S1. (2) We excluded cases where there was insufficient taxonomic sampling, such as the *betatetravirus* overlap, since the *betatetravirus* genus comprises only ones species.
(DOCX)

Table S3 **Low complexity and predicted structural disorder in proteins encoded by overlapping genes in human T-lymphotropic virus 1 (HTLV1).** (1) Predictions of low sequence complexity were made with SEG with parameters 45/3.75/3.4. (2) Predictions of structural disorder were made with MetaPrDOS (see Material and Methods).
(DOC)
