# Supplementary material for: Viral Proteins Originated De Novo by Overprinting Can Be Identified by Codon Usage: Application to the “Gene Nursery” of Deltaretroviruses
Source: PLoS Comput Biol. 2013 Aug 15;9(8):e1003162. doi: 10.1371/journal.pcbi.1003162 (PMC3744397; doi:10.1371/journal.pcbi.1003162)
Supplement: Table S1 — Taxonomic distribution of the ancestral and de novo frames of the benchmark dataset. (DOC) [file pcbi.1003162.s001.doc]

**Supplementary Table 1**

**Taxonomic distribution of the ancestral and *de novo* frames of the benchmark dataset**

A frame is identified as being ancestral if it has homologs in at least two viral families whereas the other frame (the *de novo* frame) has homologs only in at most one of these families (see Material and Methods). We present the taxonomic distribution of homologs, and backing evidence, for each ancestral and *de novo* frame respectively in the left and right part of each table. The taxonomic ranking is as follows: “species” are members of a “genus” (plural “genera”), which are contained within “families”.

Most ancestral frames belong to an existing PFAM family, identified by using HHpred (see Material and Methods). In some cases, this PFAM family itself belongs to a larger PFAM “Clan” (a grouping of homologous families), indicated here. In some other cases, no PFAM clan exists, but we identified homologous PFAM families by profile-profile comparison using HHalign . In cases of proteins for which no PFAM family could be identified, but for which a 3D structure was available, we identified distant homologs by structural comparisons, and we indicated the PFAM family or clan to which they belong. The taxonomic distribution indicated here is that given by PFAM. In many cases, it would have been highly impractical to list the taxonomic distribution in full, and we only mention the corresponding number of viral families; the reader can easily visualize the taxonomic distribution of each PFAM family by clicking on the “species” tab of this family in the PFAM website http://pfam.sanger.ac.uk.

In cases where the above approaches did not detect homology to a PFAM family, we carried out extensive efforts to identify homologs by using a variety of other approaches (see Material and Methods). The taxonomic distribution presented here is the result of these efforts.

We found 6 classes of ancestral frames that have been overprinted repeatedly (presumably independently) by *de novo* frames, and we present the dataset in 6 subtables grouped accordingly, with corresponding bibliographical references for each class (e.g. jelly-roll fold capsids; 30K movement proteins; etc). The remaining ancestral frames, each overprinted only once in our dataset, are presented in the final subtable.

|  | Ancestral frame:  Jelly-roll fold capsid proteins from icosahedral viruses | | | | | | *De novo* frame | | | |
| --- | --- | --- | --- | --- | --- | --- | --- | --- | --- | --- |
| **Genus** | **Name** | **Accession number** | **How remote homologs were identified** | **Corresponding PFAM clan** | **Taxonomic distribution** | **Name** | | **Accession number** | **PFAM family** | **Taxonomic distribution** |
| *Betatetra* | Capsid | [YP_025096.1](http://www.ncbi.nlm.nih.gov/protein/48697171) | Match to PFAM family Peptidase_A21 | Viral_ssRNA_CP | 20 viral families | Replicase  (C‑term domain) | | [NP_048059.1](http://www.ncbi.nlm.nih.gov/protein/9631280) | - | genus *betatetravirus*  (contains only one species) |
| *Aquabirna* | VP2 | [NP_047196.1](http://www.ncbi.nlm.nih.gov/protein/9630637) | Match to PFAM family Birna_VP2 |  |  | VP5 | | [NP_047195.1](http://www.ncbi.nlm.nih.gov/protein/9630636) | Birna_VP5 | genera *aquabirnavirus* and *avibirnavirus* (family *Birnaviridae*) |
| *Noro* | Capsid VP1 | [YP_720002.1](http://www.ncbi.nlm.nih.gov/protein/113478396) | Match to PFAM family  Rhv |  |  | VF1 | | [YP_006390081.1](http://www.ncbi.nlm.nih.gov/protein/388683808) | - | some species in genus *norovirus* |
| *Apara* | Capsid | [YP_001040003.2](http://www.ncbi.nlm.nih.gov/protein/389604314) | Match to PFAM family  Rhv |  |  | Pog | | [YP_006390080.1](http://www.ncbi.nlm.nih.gov/protein/388604567) | - | genus *aparavirus* |
| *Dependo* | VP2 | [YP_680427.1](http://www.ncbi.nlm.nih.gov/protein/110645921) | Structural similarity to jelly-roll fold capsids |  |  | AAP | | [YP_004030758.1](http://www.ncbi.nlm.nih.gov/protein/312281378) | - | genus *dependovirus* |
| *Parvo* | Capsid 2 | [NP_757372.1](http://www.ncbi.nlm.nih.gov/protein/26335933) | Structural similarity to jelly-roll fold capsids |  |  | SAT | | [YP_006355433.1](http://www.ncbi.nlm.nih.gov/protein/388262475) | - | genus *parvovirus* |
| *Machlomo* | Capsid | [NP_619722.1](http://www.ncbi.nlm.nih.gov/protein/20162542) | Match to PFAM family  Viral_coat |  |  | p31 | | [NP_619720.1](http://www.ncbi.nlm.nih.gov/protein/20162541) | - | genus *machlomovirus* |
| *Omegatetra* | Capsid | [YP_025096.1](http://www.ncbi.nlm.nih.gov/protein/48697171) | Match to PFAM family  Peptidase_A21 |  |  | p17 | | [YP_025095.1](http://www.ncbi.nlm.nih.gov/protein/48697172) | - | genus *omegatetravirus* |
| *Luteo* | Coat P3 | [NP_620067.1](http://www.ncbi.nlm.nih.gov/protein/20219028) | Match to PFAM family  Luteo_coat |  |  | P4 | | [NP_620068.1](http://www.ncbi.nlm.nih.gov/protein/20219029) | Luteo_Vpg | genuses *luteovirus* and *polerovirus* (family *Luteoviridae*) |
| *Carmo* | Capsid | [NP_619676.1](http://www.ncbi.nlm.nih.gov/protein/20153394) | Match to PFAM family  Viral_coat |  |  | p25 | | [NP_619677.1](http://www.ncbi.nlm.nih.gov/protein/20153393) | - | species *hibiscus ringspot chlorotic virus* |

|  | Ancestral frame:  Plant virus movement proteins of the 30K type | | | | | *De novo* frame | | | |
| --- | --- | --- | --- | --- | --- | --- | --- | --- | --- |
| **Genus** | **Name** | **Accession number** | **How remote homologs were identified** | **Corresponding homologous PFAM families** | **Taxonomic distribution** | **Name** | **Accession number** | **PFAM family** | **Taxonomic distribution** |
| *Capillo* | MP | [NP_044336.1](http://www.ncbi.nlm.nih.gov/protein/9629175) | Match to PFAM family MP | 3A, Gemini_BL1, TBSV_P22, Tombus_movement, Tenui_NS4, Bromo_MP | ≥ 10 viral families | Polyprotein | [NP_044335.1](http://www.ncbi.nlm.nih.gov/protein/9629174) | - | genus *capillovirus* |
| *Tombus* | p22 | [NP_062900.1](http://www.ncbi.nlm.nih.gov/protein/9790332) | Match to PFAM family TBSV_P22 |  |  | p19 | [NP_062901.1](http://www.ncbi.nlm.nih.gov/protein/9790333) | Tombus_P19 | genera *tombusvirus* and *aureusvirus* |
| *Umbra* | ORF4 | [NP_733850.1](http://www.ncbi.nlm.nih.gov/protein/24943166) | Match to PFAM family 3A |  |  | ORF3 | [NP_733849.1](http://www.ncbi.nlm.nih.gov/protein/24943163) | Umbravirus_LDM | genus *umbravirus* |

|  | Ancestral frame:  Coat protein of filamentous viruses | | | | | *De novo* frame | | | |
| --- | --- | --- | --- | --- | --- | --- | --- | --- | --- |
| **Genus** | **Name** | **Accession number** | **How remote homologs were identified** | **Corresponding homologous PFAM families** | **Taxonomic distribution** | **Name** | **Accession number** | **PFAM family** | **Taxonomic distribution** |
| *Mandari* | Capsid | [NP_203557.1](http://www.ncbi.nlm.nih.gov/protein/15426411) | Match to PFAM family  Flexi_CP | Poty_coat, Closter_coat, Tricho_coat, Viral_Hsp90 | Families *Alphaflexiviridae, Betaflexiviridae, Gammaflexiviridae, Potyviridae, Closteroviridae* | NABP  (N-term domain) | [NP_203558.1](http://www.ncbi.nlm.nih.gov/protein/15426412) | - | genus *mandarivirus*  (contains only one species) |
| *Tricho* | Coat | [NP_040553.1](http://www.ncbi.nlm.nih.gov/protein/9626208) | Match to PFAM family  Tricho_coat |  |  | MP (C-term domain) | [NP_040552.1](http://www.ncbi.nlm.nih.gov/protein/9626207) | - | genus *trichovirus* |

|  | Ancestral frame:  Viral Triple Gene Block 2 proteins | | | | | *De novo* frame | | | |
| --- | --- | --- | --- | --- | --- | --- | --- | --- | --- |
| **Genus** | **Name** | **Accession number** | **How remote homologs were identified** | **Corresponding PFAM clan** | **Taxonomic distribution** | **Name** | **Accession number** | **PFAM family** | **Taxonomic distribution** |
| *Potex* | TGBp2 | [NP_042697.1](http://www.ncbi.nlm.nih.gov/protein/9628110) | Match to PFAM family Plant_vir_prot | - | Family *Virgaviridae*, some genera in families *Alphaflexiviridae* and *Betaflexiviridae*, and unclassified genus *Benyvirus* | TGBp3  (N-term domain) | [NP_042698.1](http://www.ncbi.nlm.nih.gov/protein/9628111) | - | Species *cassava common mosaic virus* |
| *Hordei* | TGBp2 | [NP_604488.1](http://www.ncbi.nlm.nih.gov/protein/19744925) | Match to PFAM family Plant_vir_prot |  |  | TGBp3  (N-term domain) | [NP_604489.1](http://www.ncbi.nlm.nih.gov/protein/19744926) | Viral_Beta_CD | genera *hordeivirus*, *pecluvirus*, *pomovirus* in family *Virgaviridae* |

|  | Ancestral frame:  Rolling circle replication endonucleases | | | | | *De novo* frame | | | | |  |
| --- | --- | --- | --- | --- | --- | --- | --- | --- | --- | --- | --- |
| **Genus** | **Name** | **Accession number** | **How remote homologs were identified** | **Corresponding PFAM clan** | **Taxonomic distribution** | | **Name** | **Accession number** | **PFAM family** | **Taxonomic distribution** | |
| *Begomo* | AL1 | [NP_077100.1](http://www.ncbi.nlm.nih.gov/protein/13186217) | Match to PFAM family Gemini_AL1 | Rep | Families *Geminiviridae,* *Circoviridae*, *Nanoviridae, Polyomaviridae* | | AC4 | [NP_077101.1](http://www.ncbi.nlm.nih.gov/protein/13186218) | Gemini_C4 | genera *begomovirus*, *curtovirus*, *topocuvirus* (family *Geminiviridae*) | |
| *Brevidenso* | NS1 | [NP_694827.1](http://www.ncbi.nlm.nih.gov/protein/23334603) | Match to PFAM family Parvo_NS1 |  |  |  | NS2 | [NP_694828.1](http://www.ncbi.nlm.nih.gov/protein/23334604) | - | genus *brevidensovirus* | |
| *Denso* | NS1 | NP_874381.1 | Conservation of catalytic residues |  |  |  | NS2 | [YP_164340.1](http://www.ncbi.nlm.nih.gov/protein/23334623) | - | genera *densovirus*, *iteravirus*, *pefudensovirus* (family *Parvoviridae*) | |

|  | Ancestral frame:  Viral DNA-or RNA-dependent RNA polymerases | | | | | *De novo* frame | | | |
| --- | --- | --- | --- | --- | --- | --- | --- | --- | --- |
| **Genus** | **Name** | **Accession number** | **How remote homologs were identified** | **Corresponding PFAM clan** | **Taxonomic distribution** | **Name** | **Accession number** | **PFAM family** | **Taxonomic distribution** |
| *Orthohepadna* | Pol | [NP_647604.2](http://www.ncbi.nlm.nih.gov/protein/57021117) | Match to PFAM family RVT_1 | RdRP | ≥ 40 viral families | L | [YP_355333.1](http://www.ncbi.nlm.nih.gov/protein/77680739) | - | Family *Orthohepadnaviridae* |
| *Alphacarmotetra* | p104 | [YP_003620397.1](http://www.ncbi.nlm.nih.gov/protein/295984042) | Match to PFAM family Rdrp_3 |  |  | p130 | [YP_003620396.1](http://www.ncbi.nlm.nih.gov/protein/295984041) | - | Genus a*lphacarmotetravirus* |

|  | Ancestral frame: isolated cases | | | | | *De novo* frame | | | | |
| --- | --- | --- | --- | --- | --- | --- | --- | --- | --- | --- |
| **Genus** | **Name (function)** | **Accession number** | **How remote homologs were identified** | **Corresponding PFAM clan** | **Taxonomic distribution** | | **Name** | **Accession number** | **PFAM family** | **Taxonomic distribution** |
| *Orthohepadna* | Pol (RNAse H domain) | [NP_647604.2](http://www.ncbi.nlm.nih.gov/protein/57021117) | HHpred match to structure of Moloney murine leukemia virus RNAse H domainstructure (PDB 1rw3), and conservation of catalytic residues | RNAse_H | ≥40 families | | protein X | [NP_647606.1](http://www.ncbi.nlm.nih.gov/protein/21326587) | X | genus *orthohepadnavirus* |
| *Carmo* | p28  (co-factor of the polymerase of *Tombusviridae*-like viruses) | [NP_619671.1](http://www.ncbi.nlm.nih.gov/protein/20153389) | Match to PFAM family Tombus_P33 | No PFAM clan but PFAM families Tombus_P33, Luteo_P1_P2 are related | Family *Tombusviridae*, Genus *luteovirus* within family *Luteoviridae* | | p23 | [NP_619673.1](http://www.ncbi.nlm.nih.gov/protein/20153390) | - | Species *hibiscus ringspot chlorotic virus* |
| *Tymo* | Replicase (Methyltransferase-Guanylyltransferase domain of Alphavirus-like viruses) | [NP_663297.1](http://www.ncbi.nlm.nih.gov/protein/21686952) | Match to PFAM family Vmethyltransf | - | ≥10 families | | MP | [NP_663296.1](http://www.ncbi.nlm.nih.gov/protein/21686951) | Tymo_45kd_70kd | Genus *tymovirus* |
| *Orthobunya* | N (nucleoprotein) | [NP_047213.1](http://www.ncbi.nlm.nih.gov/protein/9630661) | Match to PFAM family Bunya_nucleocap | No PFAM clan but PFAM families Bunya_nucleocap,  Tenui_N, Phlebovirus_N, Tospo_nucleocap are related | Family *Bunyaviridae* (except genera *nairovirus* and *hantavirus*), and unassigned genus *tenuivirus* | | NSs | [NP_047214.1](http://www.ncbi.nlm.nih.gov/protein/9630662) | Bunya_NS-S | Genus *orthobunyavirus* |
| *Gyro* | VP2 | [NP_056773.1](http://www.ncbi.nlm.nih.gov/protein/9626430) | Match to PFAM family TT_ORF2 | - | Family *Anelloviridae*,  Genus *cytomegalovirus* in family *Herpesviridae* | | Apoptin | [NP_056774.1](http://www.ncbi.nlm.nih.gov/protein/9626431) | CAV_VP3 | Genus *gyrovirus* |

1. Biegert A, Mayer C, Remmert M, Soding J, Lupas AN (2006) The MPI Bioinformatics Toolkit for protein sequence analysis. Nucleic Acids Res 34: W335-339.

2. Dolja VV, Koonin EV (1991) Phylogeny of Capsid Proteins of Small Icosahedral Rna Plant-Viruses. Journal of General Virology 72: 1481-1486.

3. Abrescia NG, Bamford DH, Grimes JM, Stuart DI (2012) Structure unifies the viral universe. Annu Rev Biochem 81: 795-822.

4. Melcher U (2000) The '30K' superfamily of viral movement proteins. J Gen Virol 81: 257-266.

5. Yang SQ, Wang T, Bohon J, Gagne MEL, Bolduc M, et al. (2012) Crystal Structure of the Coat Protein of the Flexible Filamentous Papaya Mosaic Virus. Journal of Molecular Biology 422: 263-273.

6. Morozov SY, Solovyev AG (2003) Triple gene block: modular design of a multifunctional machine for plant virus movement. Journal of General Virology 84: 1351-1366.

7. Hickman AB, Ronning DR, Kotin RM, Dyda F (2002) Structural unity among viral origin binding proteins: Crystal structure of the nuclease domain of adeno-associated virus Rep. Molecular Cell 10: 327-337.

8. Bruenn JA (2003) A structural and primary sequence comparison of the viral RNA-dependent RNA polymerases. Nucleic Acids Research 31: 1821-1829.

9. Malik HS, Eickbush TH (2001) Phylogenetic analysis of ribonuclease H domains suggests a late, chimeric origin of LTR retrotransposable elements and retroviruses. Genome Res 11: 1187-1197.

10. Rozanov MN, Koonin EV, Gorbalenya AE (1992) Conservation of the putative methyltransferase domain: a hallmark of the 'Sindbis-like' supergroup of positive-strand RNA viruses. J Gen Virol 73 ( Pt 8): 2129-2134.
