# Supplementary material for: Viral Proteins Originated De Novo by Overprinting Can Be Identified by Codon Usage: Application to the “Gene Nursery” of Deltaretroviruses
Source: PLoS Comput Biol. 2013 Aug 15;9(8):e1003162. doi: 10.1371/journal.pcbi.1003162 (PMC3744397; doi:10.1371/journal.pcbi.1003162)
Supplement: Table S2 — Examination of possible confounding factors of codon usage analysis: relative frame of de novo genes compared to ancestral genes, and relative age of de novo frames. (1) We used taxonomic distribution as a very approximate, empirical proxy to estimate comparative ages of the overlaps. De novo frames found only in one species are considered “young” (provided there are several species in the genus considered, see note 2 below); overlaps found in more than one species but less than one genus are considered of “Intermediate” age, and overlaps found in more than one genus are considered “old”. The taxonomic distribution of de novo frames is taken from Supplementary Table S1. (2) We excluded cases where there was insufficient taxonomic sampling, such as the betatetravirus overlap, since the betatetravirus genus comprises only ones species. (DOCX) [file pcbi.1003162.s002.docx]

**Supplementary Table 2**

**Examination of possible confounding factors of codon usage analysis: relative frame of *de novo* genes compared to ancestral genes, and relative age of de novo frames.**

| Genus | Ancestral frame | *De novo frame* | P< | Relative frame | “relative age” of novel frame according to taxonomic distribution^(1)^ |
| --- | --- | --- | --- | --- | --- |
| *Noro* | Capsid | VF1 | 0.001 | +1 | Intermediate |
| *Omegatetra* | Capsid | p17 | 0.005 | +1 | Intermediate |
| *Dependo* | VP2 | AAP | 0.005 | +1 | Intermediate |
| *Carmo* (replicase/p23) | Replicase | p23 | 0.005 | +1 | Young |
| *Luteo* | P5 | P4 | 0.005 | +1 | Old |
| *Tymo* | Replicase | MP | 0.01 | +2 | Intermediate |
| *Aquabirna* | VP2 | VP5 | 0.01 | +2 | Old |
| *Capillo* | MP | Replicase | 0.025 | +1 | Intermediate |
| *Mandari* | Capsid | NABP | 0.025 | +2 | not applicable^(2)^ |
| *Carmo* (capsid/p25) | Capsid | p25 | 0.025 | +1 | Young |
| *Betatetra* | Capsid | Replicase | 0.05 | +2 | not applicable^(2)^ |
| *Gyro* | VP2 | Apoptin | 0.05 | +1 | Intermediate |
| *Potex* | TGBp2 | TGBp3 | 0.05 | +1 | Young |
| *Parvo* | VP2 | SAT |  | +1 | Intermediate |
| *Begomo* | Replicase | AC4 |  | +2 | Old |
| *Orthobunya* | N | NSs |  | +1 | Intermediate |
| *Tombus* | p22 | p19 |  | +2 | Old |
| *Denso* | NS1 | NS2 |  | +1 | Old |
| *Apara* | Capsid | Pog |  | +1 | Intermediate |
| *Orthohepadna* (pol/L) | Pol | L |  | +1 | Old |
| *Umbra* | ORF4 | ORF3 |  | +2 | Intermediate |
| *Hordei* | TGBp2 | TGBp3 |  | +1 | Old |
| *Orthohepadna* (pol/X) | Pol | X |  | +2 | Intermediate |
| *Brevidenso* | NS1 | NS2 |  | +1 | Intermediate |
| *Tricho* | Capsid | MP |  | +2 | Intermediate |

(1): We used taxonomic distribution as a very approximate, empirical proxy to estimate comparative ages of the overlaps. *De novo* frames found only in one species are considered “young” (provided there are several species in the genus considered, see note 2 below); overlaps found in more than one species but less than one genus are considered of “intermediate” age, and overlaps found in more than one genus are considered “old”. The taxonomic distribution of *de novo* frames is taken from Supplementary Table S1

(2): We excluded cases where there was insufficient taxonomic sampling, such as the *betatetravirus* overlap, since the *betatetravirus* genus comprises only ones species.
