# Supplementary material for: Viral Proteins Originated De Novo by Overprinting Can Be Identified by Codon Usage: Application to the “Gene Nursery” of Deltaretroviruses
Source: PLoS Comput Biol. 2013 Aug 15;9(8):e1003162. doi: 10.1371/journal.pcbi.1003162 (PMC3744397; doi:10.1371/journal.pcbi.1003162)
Supplement: Table S3 — Low complexity and predicted structural disorder in proteins encoded by overlapping genes in human T-lymphotropic virus 1 (HTLV1). (1) Predictions of low sequence complexity were made with SEG with parameters 45/3.75/3.4. (2) Predictions of structural disorder were made with MetaPrDOS (see Material and Methods). (DOC) [file pcbi.1003162.s003.doc]

**Supplementary Table S3**

**Low complexity and predicted structural disorder in proteins encoded by overlapping genes in human T-lymphotropic virus 1 (HTLV1).**

| **Protein** | **Regions with low sequence complexity^1^ (aa)** | **Regions predicted disordered^2^**  **(aa)** |
| --- | --- | --- |
| p12 | 2-99 | - |
| p30 | 3-137, 145-235 | 80-149, 194-214, 229-241 |
| Rex | 45-189 | 1-33, 70-190 |
| HBZ | 18-195 | 29-181 |
| Tax | - | 78-100, 324-353 |

(1) Predictions of low sequence complexity were made with SEG with parameters

45/3.75/3.4

(2) Predictions of structural disorder were made with MetaPrDOS .

1. Wootton JC (1994) Nonglobular Domains in Protein Sequences - Automated Segmentation Using Complexity-Measures. Computers & Chemistry 18: 269-285.

2. Ishida T, Kinoshita K (2008) Prediction of disordered regions in proteins based on the meta approach. Bioinformatics 24: 1344-1348.
